# Supplementary material for: A machine learning model for identifying patients at risk for wild-type transthyretin amyloid cardiomyopathy
Source: Nat Commun. 2021 May 11;12:2725. doi: 10.1038/s41467-021-22876-9 (PMC8113237; doi:10.1038/s41467-021-22876-9)
Supplement: Supplementary file 1 — Supplementary Information [file 41467_2021_22876_MOESM1_ESM.pdf]

SUPPLEMENTARY INFORMATION

A Machine Learning Model for Identifying Patients at Risk for Wild-Type Transthyretin Amyloid Cardiomyopathy

Ahsan Huda[1], Adam Castaño[1], Anindita Niyogi[1], Jennifer Schumacher[1], Michelle Stewart[1], Marianna Bruno[1], Mo Hu[2], Faraz S. Ahmad[2], Rahul C. Deo[3], Sanjiv J. Shah[2]

[1]Pfizer Inc, New York, NY
[2]Northwestern University Feinberg School of Medicine, Chicago, IL
[3]Brigham and Women's Hospital, Boston, MA

SUPPLEMENTARY METHODS

Cardiac amyloidosis diagnostic coding

Cardiac amyloidosis is an umbrella diagnosis code that includes any condition that results in deposition of amyloid fibrils into the myocardium. The 2 main types of cardiac amyloidosis are amyloidogenic transthyretin cardiomyopathy (ATTR-CM) and primary amyloidogenic light chain (AL) cardiac amyloidosis. ATTR-CM is further subdivided into hereditary and sporadic ("wild-type") forms. When physicians code for the diagnosis of ATTR-CM, they may use the general ICD code ("cardiac amyloidosis"), or in cases of ATTR-CM, they may use more specific terms (e.g., "wild-type ATTR-CM"). Our initial machine learning models were built on the specific diagnostic code for wild-type ATTR-CM. However, given the fact that many physicians may use the more general term of "cardiac amyloidosis" to describe ATTR-CM in diagnostic coding, we also examined the performance of our ATTR-CM machine learning algorithm on the cardiac amyloidosis diagnosis. In addition, we created a second machine learning model for the cardiac amyloidosis ICD code.

The diagnosis of cardiac amyloidosis typically involves a combination of echocardiography and/or cardiac MRI; laboratory testing (for AL amyloidosis); nuclear scintigraphy (for ATTR-CM); genetic testing (for ATTR-CM); and, in some cases, tissue biopsy (for confirmation of the diagnosis). Although ATTR-CM is a specific diagnosis, it can be coded as either ATTR-CM or cardiac amyloidosis. A machine learning model for the more general diagnosis of "cardiac amyloidosis" would still be valuable for clinicians as it would provide impetus to do further testing to confirm the diagnosis of cardiac amyloidosis with subsequent determination of the specific type of cardiac amyloidosis.

Cardiac amyloidosis machine learning model development

The machine learning model that we created for wild-type ATTR-CM was based on the specific ICD code for wild-type ATTR-CM. Cases in the derivation dataset (IQVIA) were required to have the wild-type ATTR-CM ICD code, and controls were only those who did not have either the wild-type ATTR-CM or cardiac amyloidosis ICD codes. The same is true for the Optum validation dataset. We also examined how this model (trained on the wild-type ATTR-CM diagnostic code) worked for the more general diagnostic term of cardiac amyloidosis. We performed this validation in IQVIA, Optum, and the Northwestern validation datasets, which were based on cases with cardiac amyloidosis and controls without cardiac amyloidosis. We also created a *de novo* machine learning model specifically for cardiac amyloidosis. We started with IQVIA cases with cardiac amyloidosis and IQVIA controls without cardiac amyloidosis to derive the machine learning model for the cardiac amyloidosis diagnostic code, in the exact same way that we derived the wild-type ATTR-CM model. We then tested this cardiac amyloid-specific model in the validation cohorts.

Supplementary Table 1. Data Sources and Cohort Characteristics for ATTR-CM Machine Learning Model Training, Internal Testing, Validation, and External Testing (Prediction) Analyses

Stage	Cohort	Data source	Cases[a]	Controls[a,b]
**Training and internal testing**	1	IQVIA[c] (medical claims data)	**ATTRwt-CM + HF (n=1,071)**<br>• Patients with ATTRwt-CM code (E85.82) + HF code<br>• Training set:[d] 80% of patients<br>• Test set: 20% of patients	**Non-amyloid HF (n=1,071)**<br>• Patients with a HF code but none of the amyloidosis codes<br>• Training set: [d] 80% of patients<br>• Test set: 20% of patients
**Validation**	2	Optum[e] (EHR data)	**ATTRwt-CM + HF (n=173)**<br>• Patients with ATTRwt-CM code (E85.82) + HF code	**Non-amyloid HF (n=173)**<br>• Patients with a HF code but none of the amyloidosis codes
	3	IQVIA[c] (medical claims data)	**CA + HF (n=7,296)**<br>• Patients with organ-limited amyloidosis code (E85.4) + HF code but not ATTRwt-CM, BC, LC, ESRD, CAA, or ICH diagnoses	**Non-amyloid HF (n=7,296)**<br>• Patients with a HF code but none of the amyloidosis codes
	4	Optum[e] (EHR data)	**CA + HF (n=1,943)**<br>• Patients with organ-limited amyloidosis code (E85.4) + HF code but not BC, LC, ESRD, CAA, or ICH diagnoses	**Non-amyloid HF (n=1,943)**<br>• Patients with a HF code but none of the amyloidosis codes
**External testing (prediction)**	5	Northwestern Medicine EDW[a,f] (EHR data)	**CA + HF (n=261)**<br>• Patients with organ-limited amyloidosis code (E85.4) + HF code but not BC, LC, ESRD, CAA, or ICH diagnoses	**Non-amyloid HF (n=39,393)**<br>• Patients with a HF code but none of the amyloidosis codes

Codes are International Classification of Diseases, 10th version, codes.
[a]Cases and controls in all cohorts were ≥50 years of age; [b]All controls were 1:1 propensity matched by age, gender, number of encounters, and number of years of available data; [c]Date range: 2008 to 2018; dataset: n>300 million; [d]A classification model for training and testing was developed using a Random Forest algorithm; [e]Date range: 2008 to 2018; dataset: n=88 million; [f]Date range: 2009 to 2019.
ATTRwt, wild-type transthyretin amyloidosis; BC, blood cancer; CA, cardiac amyloidosis; CAA, cerebral amyloid angiopathy; EDW, Enterprise Data Warehouse; ESRD, end-stage renal disease; ICH, intracranial hemorrhage; LC, immunoglobulin light chain amyloidosis; pts, patients.

Supplementary Table 2. Comparison of Statistical Model Performance: Logistic Regression vs. Random Forest vs. XGBoost in the IQVIA Derivation Cohort (Cohort 1)

Model	Model characteristics	Features included in the models			Nested cross-validation (mean [SD])
		ICD-10 codes (N=1875 features)	ICD-10 short description (N=1199 features)	Phenotypes derived from ICD codes (N=744 features)	
Random Forest	Precision	0.86	0.86	0.88	0.84 (0.02)
	Recall	0.87	0.86	0.84	0.83 (0.02)
	F1 score	0.87	0.86	0.86	0.83 (0.02)
	Accuracy	0.87	0.85	0.86	0.83 (0.01)
	AUROC	0.93	0.93	0.93	0.92 (0.01)
Logistic Regression	Precision	0.82	0.84	0.87	0.85 (0.02)
	Recall	0.89	0.83	0.88	0.81 (0.02)
	F1 score	0.86	0.83	0.87	0.83 (0.01)
	Accuracy	0.85	0.83	0.87	0.84 (0.01)
	AUROC	0.91	0.90	0.92	0.92 (0.01)
XGBoost	Precision	0.83	0.84	0.86	0.84 (0.02)
	Recall	0.84	0.89	0.79	0.83 (0.02)
	F1 score	0.84	0.86	0.83	0.83 (0.02)
	Accuracy	0.83	0.85	0.83	0.84 (0.02)
	AUROC	0.90	0.93	0.90	0.91 (0.01)

*ICD = International Classification of Disease; SD = standard deviation; precision = positive predictive value; recall = sensitivity; F1 score = harmonic mean of precision and recall; AUROC = area under the receiver operating characteristic curve.

Supplementary Table 3. Prediction of Cardiac Amyloidosis in the Northwestern Medicine Enterprise Data Warehouse Heart Failure Cohort (Age ≥ 50 years, cardiac amyloidosis Random Forest model)

**Probability cut-off = >0.50**							
		**Predicted**		Sensitivity	63.6%	Accuracy	85.3%
		**CA**	**Non-CA HF**	Specificity	85.5%	LR+	4.38
**Actual**	**CA**	166	95	PPV	2.8%	LR-	0.43
	**Non-CA HF**	5716	33677	NPV	99.7%		
**Probability cut-off = >0.55**							
		**Predicted**		Sensitivity	50.6%	Accuracy	92.6%
		**CA**	**Non-CA HF**	Specificity	92.9%	LR+	7.13
**Actual**	**CA**	132	129	PPV	4.5%	LR-	0.53
	**Non-CA HF**	2794	36599	NPV	99.6%		
**Probability cut-off = >0.60**							
		**Predicted**		Sensitivity	36.0%	Accuracy	96.7%
		**CA**	**Non-CA HF**	Specificity	97.1%	LR+	12.37
**Actual**	**CA**	94	167	PPV	7.6%	LR-	0.66
	**Non-CA HF**	1147	38246	NPV	99.6%		
**Probability cut-off = >0.65**							
		**Predicted**		Sensitivity	22.2%	Accuracy	98.5%
		**CA**	**Non-CA HF**	Specificity	99.0%	LR+	21.78
**Actual**	**CA**	58	203	PPV	12.6%	LR-	0.79
	**Non-CA HF**	402	38991	NPV	99.5%		
**Probability cut-off = >0.70**							
		**Predicted**		Sensitivity	11.5%	Accuracy	99.1%
		**CA**	**Non-CA HF**	Specificity	99.7%	LR+	39.37
**Actual**	**CA**	30	231	PPV	20.7%	LR-	0.89
	**Non-CA HF**	115	39278	NPV	99.4%		
**Probability cut-off = >0.75**							
		**Predicted**		Sensitivity	4.2%	Accuracy	99.3%
		**CA**	**Non-CA HF**	Specificity	99.9%	LR+	72.18
**Actual**	**CA**	11	250	PPV	32.4%	LR-	0.96
	**Non-CA HF**	23	39370	NPV	99.4%		

*CA = cardiac amyloidosis; HF = heart failure; PPV = positive predictive value; NPV = negative predictive value; LR+ = positive likelihood ratio; LR- = negative likelihood ratio.

Supplementary Table 4. Top Cardiac and Non-Cardiac Phenotypes Predictive of Wild-type ATTR Cardiomyopathy

Phenotype	Prevalence in wild-type ATTR cardiomyopathy	Prevalence in non-amyloid heart failure	Odds ratio (95% CI)	P-value
Secondary intrinsic cardiomyopathies	70.4%	4.3%	53.0 (38.2-74.9)	3.3×10^{-251}
Primary intrinsic cardiomyopathies	70.3%	27.9%	6.1 (5.0-7.4)	4.9×10^{-88}
Carpal tunnel	31.9%	7.8%	5.5 (4.2-7.2)	1.9×10^{-46}
Pericardial effusion/pericarditis	18.5%	4.2%	5.2 (3.7-7.4)	9.5×10^{-27}
Atrial flutter	49.4%	18.8%	4.2 (3.5-5.2)	1.5×10^{-51}
HFpEF	71.7%	40.4%	3.7 (3.1-4.5)	9.7×10^{-49}
HFrEF	73.6%	44.2%	3.5 (2.9-4.2)	5.7×10^{-44}
Synovitis and tenosynovitis	20.9%	7.2%	3.4 (2.6-4.6)	2.4×10^{-20}
Non-rheumatic heart valve disease	16.2%	5.4%	3.4 (2.5-4.7)	3.5×10^{-16}
First-degree AV block	19.7%	6.9%	3.3 (2.5-4.4)	1.3×10^{-18}
Chronic pulmonary heart disease	38.7%	16.6%	3.2 (2.6-3.9)	1.8×10^{-30}
Abnormal serum enzymes	29.3%	12.2%	3.0 (2.4-3.8)	1.0×10^{-22}
Non-specific abnormal findings on radiological examination of intrathoracic organs	17.2%	6.8%	2.8 (2.1-3.8)	1.2×10^{-13}
Cardiomegaly	65.1%	39.8%	2.8 (2.4-3.4)	7.5×10^{-32}
Ascites	15.4%	6.2%	2.8 (2.0-3.8)	4.4×10^{-12}
Paroxysmal ventricular tachycardia	26.8%	12.1%	2.6 (2.1-3.4)	8.2×10^{-18}
Fluid overload	18.1%	8.0%	2.5 (1.9-3.4)	4.1×10^{-12}
Chronic heart disease (other)	29.3%	14.7%	2.4 (1.9-3.0)	2.4×10^{-16}
Abnormal blood chemistry	44.7%	25.1%	2.4 (2.0-2.9)	1.7×10^{-21}
Bundle branch block	35.3%	18.7%	2.4 (1.9-2.9)	4.4×10^{-18}
Atrial fibrillation	72.2%	52.3%	2.4 (2.0-2.8)	2.3×10^{-21}
Long-term anticoagulation use	53.1%	32.4%	2.4 (2.0-2.8)	3.3×10^{-22}
Premature beats	29.2%	15.6%	2.2 (1.8-2.8)	4.0×10^{-14}
Paroxysmal supraventricular tachycardia	16.2%	8.3%	2.1 (1.6-2.8)	3.5×10^{-08}
Inflammatory and toxic neuropathy	22.9%	12.4%	2.1 (1.7-2.7)	2.5×10^{-10}
Pleural effusion	47.5%	30.5%	2.1 (1.7-2.5)	8.9×10^{-16}
Abnormal ECG	54.9%	37.6%	2.0 (1.7-2.4)	1.3×10^{-15}
Soft tissue disorders	27.0%	15.6%	2.0 (1.6-2.5)	1.4×10^{-10}
Hypertensive heart and/or renal disease	69.5%	53.4%	2.0 (1.7-2.4)	2.7×10^{-14}
Tricuspid valve disorder	38.4%	24.3%	1.9 (1.6-2.4)	2.4×10^{-12}
Mitral valve disorder	50.5%	34.5%	1.9 (1.6-2.3)	9.6×10^{-14}

Univariate logistic regression was used to calculate odds ratios. P-values are 2-sided. Adjustments were not made for multiple comparisons. Only phenotypes with a minimal prevalence of 15% in wild-type ATTR cardiomyopathy were included. HFpEF = heart failure with preserved ejection fraction; HFrEF = heart failure with reduced ejection fraction; AV = atrioventricular; ECG = electrocardiogram.

Supplementary Table 5. Utility of Combinations of Phenotypes Based on ICD Codes and Their Association with Wild-Type ATTR Cardiomyopathy – Test Characteristics (IQVIA dataset, Cohort 1)

Diagnosis code combinations	TP	FP	TN	FN	Accuracy	PPV	NPV	Sensitivity	Specificity	OR
**All diagnosis code combinations**	**876**	**423**	**647**	**194**	**71%**	**67%**	**77%**	**82%**	**60%**	**6.91**
Joint disorders, osteoarthrosis, pleurisy or pleural effusion, HFpEF	558	185	886	513	67%	75%	63%	52%	83%	5.21
Heart block, cardiomegaly, HFpEF	342	84	987	729	62%	80%	58%	32%	92%	5.51
Cardiomegaly, joint disorders, HFpEF	318	75	996	753	61%	81%	57%	30%	93%	5.61
Heart block, joint disorders, combined systolic + diastolic HF	307	69	1002	764	61%	82%	57%	29%	94%	5.84
AF, joint disorders, HFpEF	307	70	1001	764	61%	81%	57%	29%	93%	5.75
Carpal tunnel syndrome	285	71	1000	786	60%	80%	56%	27%	93%	5.11
AF, cardiomegaly, joint disorders, combined systolic and diastolic HF	258	61	1010	813	59%	81%	55%	24%	94%	5.25
AF, joint disorders, pleurisy or pleural effusion, combined systolic + diastolic HF	254	61	1010	817	59%	81%	55%	24%	94%	5.15
AF, heart block, CKD, pleurisy or pleural effusion, combined systolic and diastolic HF	249	56	1015	822	59%	82%	55%	23%	95%	5.49
AF, cardiomegaly, soft tissue disease, HFpEF	236	55	1016	835	58%	81%	55%	22%	95%	5.22
Heart block, cardiomegaly, joint disorders	233	50	1021	838	59%	82%	55%	22%	95%	5.68
Combined systolic and diastolic HF	230	54	1017	841	58%	81%	55%	21%	95%	5.15
AF, cardiomegaly, soft tissue disease, HFpEF	199	45	1026	872	57%	82%	54%	19%	96%	5.20
AF, CKD, pleurisy or pleural effusion, soft tissue disease, combined systolic and diastolic HF	196	42	1029	875	57%	82%	54%	18%	96%	5.49
Heart block, CKD, HFpEF	194	39	1032	877	57%	83%	54%	18%	96%	5.85
Cardiomegaly, joint disorders, soft tissue disorders, combined systolic and diastolic HF	171	38	1033	900	56%	82%	53%	16%	96%	5.17
Heart block, soft tissue disease, HFpEF	164	35	1036	907	56%	82%	53%	15%	97%	5.35
AF, heart block, joint disorders, soft tissue disorders	162	36	1035	909	56%	82%	53%	15%	97%	5.12
AF, heart block, CKD, soft tissue disease, combined systolic and diastolic HF	155	34	1037	916	56%	82%	53%	14%	97%	5.16
AF, CKD, pleurisy or pleural effusion, soft tissue disease, combined systolic and diastolic HF	154	34	1037	917	56%	82%	53%	14%	97%	5.12
AF, CKD, pleurisy or pleural effusion, combined systolic + diastolic HF	126	27	1044	945	55%	82%	52%	12%	97%	5.16

Univariate logistic regression was used to calculate odds ratios. P-values are 2-sided. Adjustments were not made for multiple comparisons.

Abbreviations: ATTR = amyloidogenic transthyretin cardiomyopathy; TP = true positive; FP = false positive; TN = true negative; FN = false negative; PPV = positive predictive value; NPV = negative predictive value; OR = odds ratio; HFpEF = heart failure with preserved ejection fraction; HF = heart failure; AF = atrial fibrillation or flutter; CKD = chronic kidney disease

Supplementary Figure 1. Derivation of the Optimal Cut-off for the Diagnosis of Wild-type ATTR Cardiomyopathy Based on Model Performance Characteristics in the IQVIA Test Dataset (Cohort 1 Test Set)

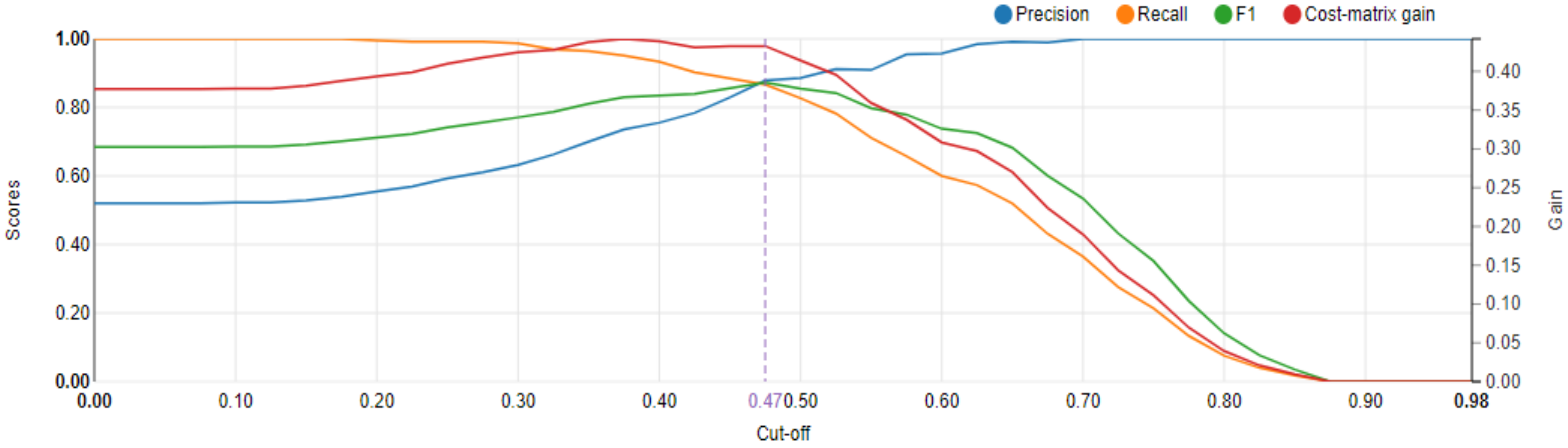

Recall = sensitivity; F1 = harmonic mean of precision and recall (sensitivity). Cost matrix gain = improvement in the cost matrix (used to specify the relative importance of accuracy for different predictions) based on varying probability cut-offs for the prediction of wild-type ATTR cardiomyopathy.

Supplementary Figure 2. Flowchart for Selection of Cases and Controls in the Northwestern External Validation Cohort (Cohort 5)

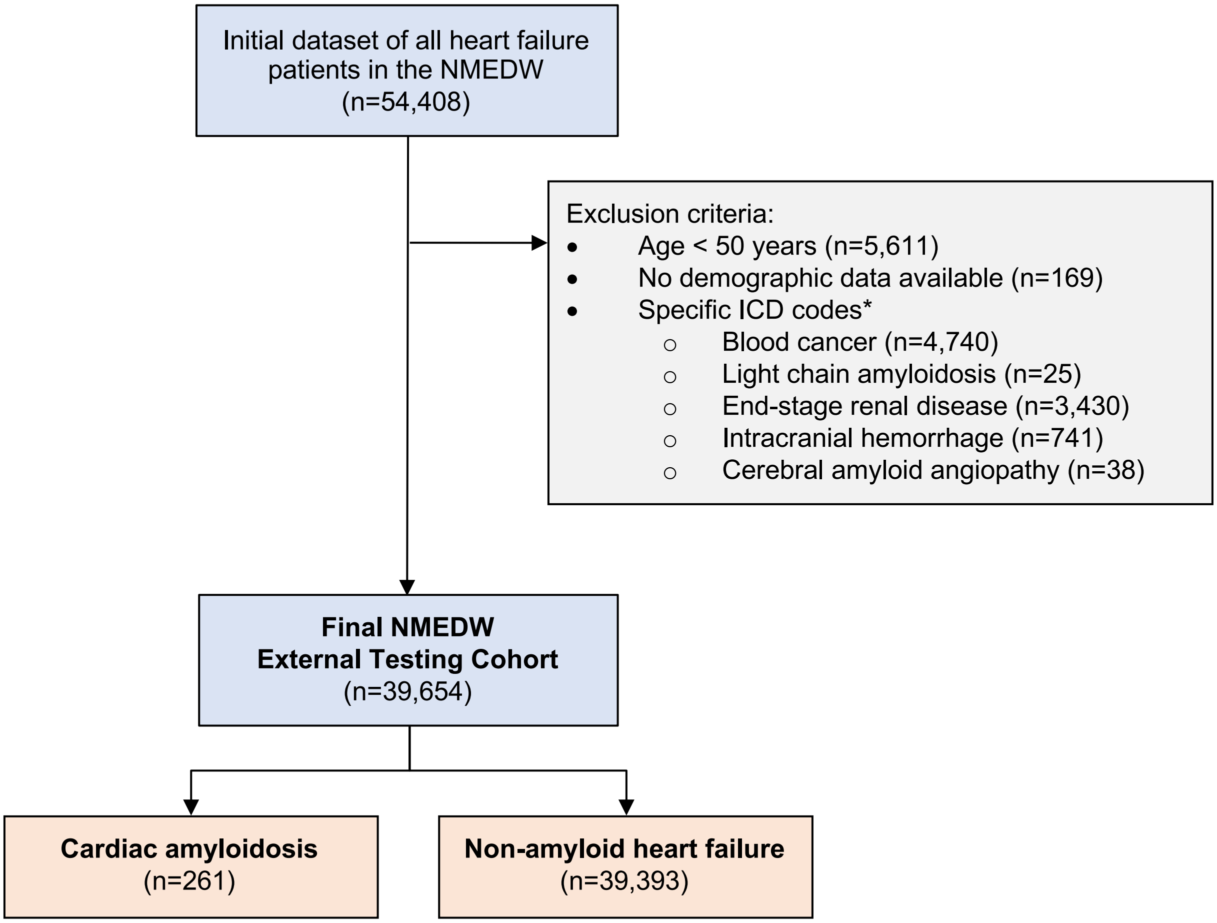

*Exclusions based on ICD codes were done sequentially using the order presented in the flowchart above (patients could have more than 1 of the exclusionary ICD diagnoses, but were only counted once based on the first diagnosis by which they were excluded). Specific ICD codes used for exclusions:

- Blood cancer: ICD-9 (200.80, 200.81, 200.82, 200.83, 200.84, 200.85, 200.86, 200.87, 200.88, 200.00, 200.01, 200.02, 200.03, 200.04, 200.05, 200.06, 200.07, 200.08, 20280, 20281, 20282, 20283, 20284, 20285, 20286, 20287, 20288, 200.51, 200.52, 200.53, 200.54, 200.55, 200.56, 200.57, 200.58, 200.50, 200.71, 200.72, 200.73, 200.74, 200.75, 200.76, 200.77, 200.78, 200.70, 203.00, 203.01, 203.02, 203.10, 203.11, 203.12, 203.80, 203.81, 203.82, 273.1, 273.2, 273.3, 273.8, 273.9); ICD-10 (C8300, C8301, C8302, C8303, C8304, C8305, C8306, C8307, C8308, C8309, C8330, C8331, C8332, C8333, C8334, C8335, C8336, C8337, C8338, C8339, C8510, C8511, C8512, C8513, C8514, C8515, C8516, C8517, C8518, C8519, C9000, C9001, C9002, C9010, C9011, C9012, C888, C9021, C9020, C9031, C9022, C9030, C880, D472, D891, E8809)
- Light chain amyloidosis: ICD-10 (E8581)
- End-stage renal disease: ICD-9 (5856); ICD-10 (N186)
- Intracranial hemorrhage: ICD-9 (431)
- Cerebral amyloid angiopathy: ICD-10 (I680)

NMEDW = Northwestern Medicine Enterprise Data Warehouse; ICD = International Classification of Diseases.
