# Supplementary material for: A machine learning model for identifying patients at risk for wild-type transthyretin amyloid cardiomyopathy
Source: Nat Commun. 2021 May 11;12:2725. doi: 10.1038/s41467-021-22876-9 (PMC8113237; doi:10.1038/s41467-021-22876-9)
Supplement: Supplementary file 3 — Reporting Summary [file 41467_2021_22876_MOESM3_ESM.pdf]

nature research

Corresponding author(s): Sanjiv J. Shah

Last updated by author(s): Jan 15, 2021

Reporting Summary

Nature Research wishes to improve the reproducibility of the work that we publish. This form provides structure for consistency and transparency in reporting. For further information on Nature Research policies, see our Editorial Policies and the Editorial Policy Checklist.

Statistics

For all statistical analyses, confirm that the following items are present in the figure legend, table legend, main text, or Methods section.

n/a	Confirmed	
[ ]	[x]	The exact sample size (*n*) for each experimental group/condition, given as a discrete number and unit of measurement
[x]	[ ]	A statement on whether measurements were taken from distinct samples or whether the same sample was measured repeatedly
[ ]	[x]	The statistical test(s) used AND whether they are one- or two-sided<br>*Only common tests should be described solely by name; describe more complex techniques in the Methods section.*
[ ]	[x]	A description of all covariates tested
[ ]	[x]	A description of any assumptions or corrections, such as tests of normality and adjustment for multiple comparisons
[ ]	[x]	A full description of the statistical parameters including central tendency (e.g. means) or other basic estimates (e.g. regression coefficient) AND variation (e.g. standard deviation) or associated estimates of uncertainty (e.g. confidence intervals)
[ ]	[x]	For null hypothesis testing, the test statistic (e.g. *F*, *t*, *r*) with confidence intervals, effect sizes, degrees of freedom and *P* value noted<br>*Give P values as exact values whenever suitable.*
[x]	[ ]	For Bayesian analysis, information on the choice of priors and Markov chain Monte Carlo settings
[x]	[ ]	For hierarchical and complex designs, identification of the appropriate level for tests and full reporting of outcomes
[x]	[ ]	Estimates of effect sizes (e.g. Cohen's *d*, Pearson's *r*), indicating how they were calculated

Our web collection on statistics for biologists contains articles on many of the points above.

Software and code

Policy information about availability of computer code

Data collection	Data sets were sourced from vendors IQVIA Inc and Optum Inc. SQL queries were used to extract data from medical claims and electronic health records data sets from IQVIA and Optum respectively. Validation in the Northwestern cohort was obtained in a de-identified fashion from the Northwestern Medicine Enterprise Data Warehouse (electronic health record data collected for clinical purposes).
Data analysis	All data analyses was performed using custom scripts and open source libraries in Python (versions 3.4 and 3.6) and R (version 3.2). The IQVIA training and validation datasets have been made available for download (see Supplementary Information). The Optum and Northwestern Medicine Enterprise Data Warehouse datasets used for this study could not be made available publicly due to data use agreements and the possibility for identification of individual patients, respectively, but will be made available to qualified investigators upon reasonable request. The code used for training and validation of the models (including instructions for use of the code and notations for the software needed to run the code) have been made available for download (see Supplementary Information). The following software packages were used within Python (versions 3.4 and 3.6): numpy, pandas, sklearn, matplotlib, and GridSearchCV. We also used the MatchIt package in R (version 3.2).

For manuscripts utilizing custom algorithms or software that are central to the research but not yet described in published literature, software must be made available to editors and reviewers. We strongly encourage code deposition in a community repository (e.g. GitHub). See the Nature Research guidelines for submitting code & software for further information.

Data

Policy information about availability of data

All manuscripts must include a data availability statement. This statement should provide the following information, where applicable:

- Accession codes, unique identifiers, or web links for publicly available datasets
- A list of figures that have associated raw data
- A description of any restrictions on data availability

All raw and processed data used in the manuscript will be made available for review.

Field-specific reporting

Please select the one below that is the best fit for your research. If you are not sure, read the appropriate sections before making your selection.

[x] Life sciences [ ] Behavioural & social sciences [ ] Ecological, evolutionary & environmental sciences

For a reference copy of the document with all sections, see nature.com/documents/nr-reporting-summary-flat.pdf

Life sciences study design

All studies must disclose on these points even when the disclosure is negative.

Sample size	We performed Monte-Carlo simulations to determine the minimal sample sizes required to adequately develop a predictive model for ATTR cardiomyopathy based on diagnosis codes from medical claims and/or electronic health record data. Using an estimated prevalence of ATTR-cardiomyopathy of 4% in patients with heart failure, we found that with 100 patients with ATTR cardiomyopathy, we would have >90% power (at significance level of 0.05) to develop an ATTR cardiomyopathy model that could differentiate between cases and controls. Each of the 5 cohorts used in this study (derivation dataset and 4 validation datasets) each included at least 100 patients with ATTR cardiomyopathy.
Data exclusions	We excluded patients age < 50 years because ATTR cardiomyopathy is a disease of older individuals with disease onset in the 7th-8th decade of life). We also excluded patients with diagnosis codes related to other (non-ATTR) forms of cardiac amyloidosis. These included blood cancers (which can case amyloidogenic light chain amyloidosis); the specific diagnosis of immunogloblulin light chain amyloidosis; end-stage renal disease (which can be associated with non-ATTR amyloidosis); intracranial hemorrage and cerebral amyloid angiopathy (which can be related to non-ATTR cerebral amyloidosis).
Replication	Reproducible data extraction, pre-processing, and modeling pipelines were created in Python. The Python packages takes in raw patient data and produces patient probabilities and metrics reported in the manuscript. The data and metrics reported in the manuscript have been independently reproduced by analytics teams at Pfizer and Northwestern University. We replicated the derived machine learning model for ATTR cardiomyopathy in 4 independent cohorts (IQVIA cardiac amyloidosis cases vs. non-amyloid heart failure controls; Optum ATTR cardiomyopathy vs. controls; Optum cardiac amyloidosis vs. controls; and Northwestern Enterprise Data Warehouse cardiac amyloidosis vs. controls).
Randomization	Randomization is not applicable to this study because it is a case-control study that used previously collected data. We did randomly select patients for the 80/20 split in the derivation dataset, with the 80% split used for model derivation and 20% split used for model testing to determine which model (e.g., logistic regression, Random Forest, XGBoost) performed best. We adjusted for covariates in the validation portion of our study.
Blinding	Blinding is not applicable for the derivation dataset because development of the machine learning prediction model for ATTR cardiomyopathy required knowledge of case/control status and diagnosis codes in order to develop the model. The 4 validation cohorts (datasets) were already in existence prior to the start of our study and thus included data that were collected blinded to the study hypothesis and the model inputs. Thus, our validation of our machine learning predictive model in 4 cohorts was done in a blinded fashion.

Reporting for specific materials, systems and methods

We require information from authors about some types of materials, experimental systems and methods used in many studies. Here, indicate whether each material, system or method listed is relevant to your study. If you are not sure if a list item applies to your research, read the appropriate section before selecting a response.

Materials & experimental systems

n/a	Involved in the study	
☒	☐	Antibodies
☒	☐	Eukaryotic cell lines
☒	☐	Palaeontology and archaeology
☒	☐	Animals and other organisms
☐	☒	Human research participants
☒	☐	Clinical data
☒	☐	Dual use research of concern

Methods

n/a	Involved in the study	
☒	☐	ChIP-seq
☒	☐	Flow cytometry
☒	☐	MRI-based neuroimaging

Human research participants

Policy information about studies involving human research participants

Population characteristics	This study was comprised of 3 parts: (1) derivation (training) and testing of various supervised statistical learning models for the diagnosis of wild-type ATTR cardiomyopathy in a large administrative medical claims dataset (IQVIA); (2) validation of the best-performing ATTR cardiomyopathy model in additional large administrative medical claims and electronic health record (EHR) datasets (IQVIA and Optum); and (3) testing of the final machine learning model in an EHR from a single, large healthcare system (Northwestern Medicine Enterprise Data Warehouse [NMEDW]) to observe the model's performance. The 3 parts of the study included 5 cohorts which were each comprised of cases and controls. Cases were defined as either ATTR cardiomyopathy (which is a specific type of cardiac amyloidosis) or the more general, umbrella term of cardiac amyloidosis. The 5 cohorts included in our study are as follows: IQVIA ATTR cardiomyopathy vs. controls; IQVIA cardiac amyloidosis vs. controls, Optum ATTR cardiomyopathy vs. controls; Optum cardiac amyloidosis vs. controls; and NMEDW cardiac amyloidosis vs. controls. The demographic and clinical characteristics of patients included in the 5 cohorts included in this study are consistent with prior studies of patients with ATTR cardiomyopathy and cardiac amyloidosis (mean age 73-78 years; >65% male; and more likely to have a history of atrial fibrillation and chronic kidney disease compared to non-amyloid heart failure controls).
Recruitment	Our study utilized 3 data sources: IQVIA, Optum, and the Northwestern Medicine Enterprise Data Warehouse (NMEDW). IQVIA and Optum are national representative medical claims and electronic health record databases, respectively. The NMEDW dataset is an electronic health records database for Northwestern Medicine, which includes Northwestern Memorial Hospital and affiliated hospitals/clinics. Cases and controls were identified retrospectively from the 3 aforementioned datasets using diagnosis codes. No patients were prospectively recruited for our study. A potential source of bias for the nationally representative databases (IQVIA and Optum) is the diagnosis coding for wild-type ATTR cardiomyopathy, which may not be used universally. Therefore, we may not have identified all patients with wild-type ATTR cardiomyopathy in these databases, thus limiting generalizability. However, we validated our machine learning predictive model in datasets that defined cases as the more general term of cardiac amyloidosis. The NMEDW database may be limited by referral bias (academic medical center) but was necessary as a validation dataset to determine the performance of the machine learning predictive model in an electronic health record dataset in a single, large healthcare system (which mimics how clinicians would use the machine learning model).
Ethics oversight	The Northwestern University Institutional Review Board approved the study protocol.

Note that full information on the approval of the study protocol must also be provided in the manuscript.
